# Fluorescence Characteristics and Lifetime Images of Photosensitizers of Talaporfin Sodium and Sodium Pheophorbide a in Normal and Cancer Cells

**DOI:** 10.3390/s150511417

**Published:** 2015-05-18

**Authors:** Kamlesh Awasthi, Kazuhito Yamamoto, Kazunari Furuya, Takakazu Nakabayashi, Liming Li, Nobuhiro Ohta

**Affiliations:** 1 Research Institute for Electronic Science (RIES), Hokkaido University, Sapporo 001-0020, Japan; E-Mail: kaawasthi@gmail.com; 2 Graduate School of Photonic Science, Chitose Institute for Science and Technology, Chitose 066-8655, Japan; E-Mails: h13st@yahoo.co.jp (K.Y.); b2102320@photon.chitose.ac.jp (K.F.); 3 Graduate School of Pharmaceutical Sciences, Tohoku University, Aoba-ku, Sendai 980-8578, Japan; E-Mail: takan@m.tohoku.ac.jp

**Keywords:** sodium pheophorbide a, talaporfin sodium, photodynamic therapy, reactive oxygen species, cancer cells, fluorescence spectrum, fluorescence lifetime

## Abstract

Fluorescence spectra and fluorescence lifetime images of talaporfin sodium and sodium-pheophorbide a, which can be regarded as photosensitizers for photodynamic therapy, were measured in normal and cancer cells. The reduction of the fluorescence intensity by photoirradiation was observed for both photosensitizers in both cells, but the quenching rate was much faster in cancer cells than in normal cells. These results are explained in terms of the excessive generation of reactive oxygen species via photoexcitation of these photosensitizers in cancer cells. The fluorescence lifetimes of both photosensitizers in cancer cells are different from those in normal cells, which originates from the different intracellular environments around the photosensitizers between normal and cancer cells.

## 1. Introduction

Photodynamic therapy (PDT) is defined as the photoinduced destruction of diseased cells such as cancer cells accumulating a photosensitizer [1,2,3]. Reactive oxygen species (ROSs) are effectively generated by exposure of visible or near-infrared light to accumulated photosensitizers, resulting in the irreversible degradation of diseased cells. ROS is a general term of reactive molecules such as singlet oxygen, superoxide anion, and radicals. The generation of ROS is mainly resulted from the conversion of molecular oxygen by reaction with the triplet state of the photosensitizer formed via photoexcitation, and ROSs attack biological substances such as nucleic acids and induce a series of physiological responses resulting in cell death. Photosensitizers are developed to be preferentially accumulated in diseased cells, which is able to induce the selective destruction of diseased cells. PDT is therefore a minimally invasive anticancer modality with low-power light energy.

PDT has a long history and the treatment of PDT has now been applied in a realistic medical setting to destruct diseased cells, such as macular degeneration and solid tumors. Several types of photosensitizers for PDT are commercially available [4]. Efficient generation of ROS and preferential accumulation to diseased cells are necessary for a photosensitizer to be used for PDT. Reduction of prolonged accumulation in normal cells is also very important to evade harmful side effects such as photodermatosis. Detailed information on photophysics and status of photosensitizers in living systems therefore allows us to develop new PDT photosensitizers applicable to curing many types of diseases.

Photosynthesizers for PDT are roughly divided into first-, second-, and third-generations, respectively [5,6]. Talaporfin sodium (TPS) derived from a chlorophyll and L-aspartic acid is called the second-generation photosensitizer that improves the efficiency of ROS generation and reduces side effects compared with hematoporphyrin derivatives called the first-generation photosensitizers [7,8,9,10,11,12]. It is noted that these second-generation photosensitizers exhibit an absorption band in the longer wavelength region that expands the penetration depth of irradiation light for exciting photosensitizers. Actually, TPS has the absorption band around 660 nm that arises from the chlorin E6 moiety. Talaporfin was approved in Japan, and has been used for the PDT treatment of cancers such as early lung cancer [13,14,15]. The water-soluble sodium salt of pheophorbide a, *i.e.*, sodium-pheophorbide a (Na-Ph-a), which is also a derivative of chlorophyll *a*, may also be regarded as the second-generation photosensitizer. Na-Ph-a has the absorption peak at ~665 nm. It has been shown that PDT using pheophorbide a shows antitumor effects as well as antibacterial properties [16,17,18]. It is noted that the generation of singlet oxygen with photoirradiation was shown both for TPS and for Na-Ph-a [19,20,21,22,23].

**Figure 1:**
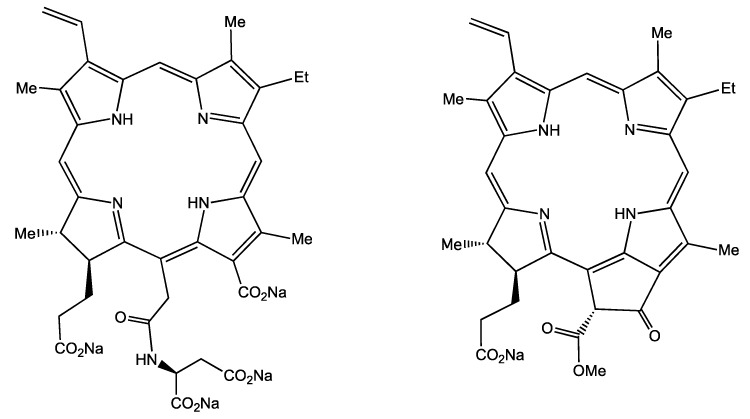
Chemical structure of talaporfin sodium (**left**) and sodium-pheophorbide a (**right**).

In the present study, we have investigated the fluorescence properties of TPS and Na-Ph-a in normal cells and in cancer cells. The molecular structures of TPS and Na-Ph-a are shown in Figure 1. Fluorescence spectrum and the efficiency of photobleaching of these photosensitizers have been compared between normal and cancer cells. Measurements of the fluorescence lifetime image (FLIM) of both photosensitizers were also performed. The difference of the fluorescence characteristics between the normal cell and the cancer cell is discussed in terms of the generation efficiency of ROS in cells. WKA rat normal fibroblast (WFB) cells and W31 cells that are H-*ras* oncogene-transfected cells from WFB [24] were used as normal and cancer cells, respectively.

## 2. Experimental Section

WFB and W31 cells were grown in a 5% CO_2_ humidified atmosphere at 37 °C in Dulbecco’s modified Eagle’s medium (DMEM, D5796, Sigma) supplemented with 2 × 10^5^ U·dm^−3^ penicillin G, 200 mg of streptomycin sulfate, and 10% fetal bovine serum (FBS) [24]. Cells were cultured on 35-mm coverslips. Commercially available TPS (Laserphyrin) was dissolved in FBS free DMEM with a concentration of 1 μM or 100 μM, and the solution was added to the cultured cells. The cells were then incubated for 30 min and were washed twice with calcium and magnesium-free phosphate buffered saline (PBS(-)). Commercially available Na-Ph-a (Chlorophyll Research Institute) was similarly dissolved in FBS free DMEM with a concentration of 10 μM, and the solution was added to the cultured cells, which were incubated for 30 min and then washed twice with PBS(-). The optical measurements were started just after the exchange of PBS(-) medium.

Measurements of FLIM of WFB and W31 cells were carried out by an inverted confocal microscope (C1, Nikon) combined with a time-correlated single photon counting (TCSPC) system (SPC-830, Becker and Hickl) [25]. The second harmonic output from a mode-locked femtosecond Ti:sapphire laser (Tsunami, Spectra Physics) was used as an excitation light source. The repetition rate of the pulse train was ~81 MHz. The fluorescence of TPS or Na-Ph-a in cells was detected by a microchannel-plate photomultiplier (R3809U, Hamamatsu). The excitation wavelength was 405 nm and the fluorescence in the wavelength region longer than 590 nm was detected. Note that the fluorescence spectra were independent of the excitation wavelength in both photosensitizers. The collected data were analyzed by SPC image software (Becker and Hickl). The observed fluorescence decays were fitted by the convolution of the instrumental response function with a multi-exponential decay. The measurement time of FLIM was less than 1 min.

Measurements of fluorescence decay profiles of TPS in solution were carried out by a homemade TCSPC system [26]. Both the excitation light source and the photomultiplier were the same as those used for FLIM, although the repetition rate of the excitation pulse was reduced to ~5.8 MHz using an electro-optic modulator (Conoptics model 350-160).

The steady-state fluorescence spectra were recorded with a fluorescence spectrometer (FP-777, JASCO). Cells were gathered into a cuvette with a 1 mm optical path, which was used for the measurements of the fluorescence spectra. Photoirradiation effects on fluorescence spectra were examined with an irradiation light intensity of ~2 mW/cm^2^.

## 3. Results and Discussion

**Figure 2:**
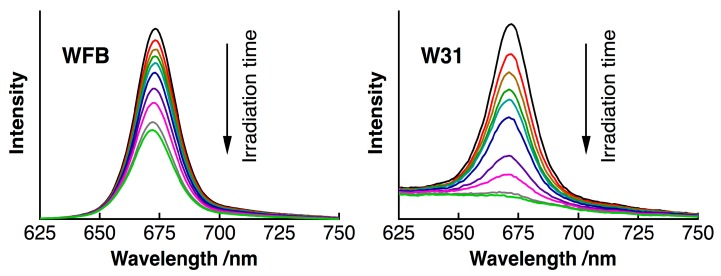
Time dependence of fluorescence spectra of TPS in WFB (**left**) and W31 (**right**) cells with irradiation of 405-nm light in air. The irradiation times were 0, 1, 2, 3, 4, 6, 10, 15, 25 and 30 min. The concentration of TPS in the culture solution was 100 μM.

**Figure 3:**
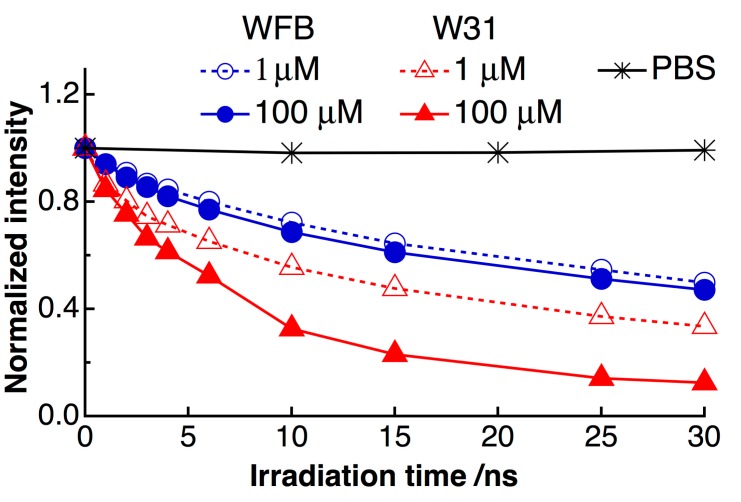
Plots of the fluorescence intensity of TPS against the irradiation time. Open (○) and filled (●) circles are WFB cells prepared at a TPS concentration of 1 μM and 100 μM, respectively. Open (△) and filled (▲) triangles are W31 cells prepared at a TPS concentration of 1 μM and 100 μM, respectively. Asterisks (*) are TPS in phosphate buffered saline (PBS) buffer. Each intensity is normalized by the intensity at 0 min.

Figure 2 shows time dependence of the fluorescence spectra of TPS in WFB (normal) and W31 (cancer) cells continuously irradiated at 405-nm light in air. The quantum yield of the fluorescence of TPS was reported to be in the order of 10^−3^ in water [10]. The peak of the fluorescence spectra was observed at around 670 nm and the difference in the peak wavelength between WFB and W31 cells was less than 2 nm just after the photoirradiation (0 min). In both cells, the temporal decrease of the fluorescence intensity due to photobleaching was clearly observed. The fluorescence intensity in W31 cells decreased more rapidly than that in WFB cells. The marked shift of the fluorescence spectrum was not observed in both cells during 15 min of photoirradiation, suggesting that photoproducts from TPS did not contribute to the observed fluorescence. Plots of the fluorescence intensity of TPS against the irradiation time are shown in Figure 3. The rate of the fluorescence quenching by photoirradiation was compared between two different concentrations of TPS, *i.e*., 1 μM and 100 μM, in the culture solution. It can be clearly seen that the quenching rate markedly increases in W31 cells with increasing concentration of TPS, while WFB cells show only the small difference in the magnitude of quenching between 1 μM and 100 μM. These differences may originate from the difference of the generation efficiency of ROS, as will be mentioned later. It was also confirmed that the fluorescence intensity of TPS in PBS buffer remained constant during 30 min of irradiation. It is mentioned that the intracellular uptake efficiencies of photosensitizers in normal and cancer cells depend on the concentration and on the incubation time of photosensitizers. The uptake of the present photosensitizers in cancer cells reaches maximum much earlier than that in normal cells [11,27]. As mentioned above, the present incubation time was 30 min, for both cancer and normal cells, after adding the photosensitizers, and the uptake efficiency in cancer cells is much higher than that in normal cells.

**Figure 4:**
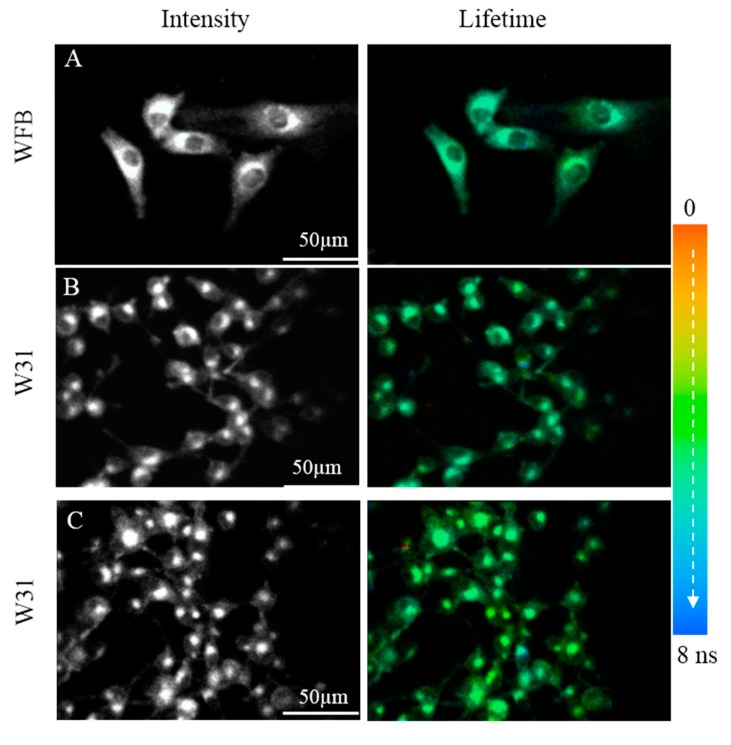
Fluorescence intensity images (**left**) and corresponding fluorescence lifetime images (**right**) of TPS in WFB cells (**A**) and in W31 cells (**B**,**C**). The image C was measured with a delay after photoirradiation rather than the image B. Pseudocolor is used for the lifetime images to show the value of the fluorescence lifetime.

The fluorescence intensity and the corresponding fluorescence lifetime images of TPS in WFB cells are shown in Figure 4A. The intensity reflects the concentration of TPS at each cellular compartment and the dull round region in WFB cells is assigned to a nucleus having a low concentration of TPS. The bright area is considered to mainly arise from lysosomes because TPS was reported to be preferentially accumulated in lysosomes [28]. Figure 4B exhibits the fluorescence intensity and lifetime images of TPS in W31 cells. The dull round region was not clearly observed in the intensity image of many W31 cells, which was thought to result from the significant damage of the cells during the measurement of FLIM. In fact, fluorescence intensity image of TPS-stained W31 cells shows the disappearance of the round region in almost all cells, when it took a rather long time to start the measurement of the image after photoirradiation (see Figure 4C). The bright area in W31 cells might be attributed to high concentrated aggregates of TPS produced during the damage of cells. These results indicate that TPS-stained W31 cells are damaged by photoirradiation much more effectively than TPS-stained WFB cells, which is consistent with a previous report [11]. The photoinduced damage of W31 cells probably results from the efficient accumulation of TPS and excessive generation of ROS inside the W31 cells, which is important to the application to PDT. The shape of the damaged cells in Figure 4C shows the morphological change due to apoptotic cell death such as cell shrinkage. In fact, excessive ROS from TPS was reported to induce apoptosis [29].

As mentioned above, the significant decrease of the fluorescence intensity of TPS with photoirradiation observed in W31 cells may be related to the efficient generation of ROS via photoexcitation. ROS may induce the destruction of cells, which is the process of PDT, but ROS also reacts to organic dyes, resulting in the change of the fluorescence intensity [30,31]. Therefore, the marked photobleaching of the fluorescence in both W31 and WFB cells can be considered to arise just from the excessive generation of ROS from TPS in W31 and WFB cells. This conclusion is consistent with the fact that the rate of the photobleaching is accelerated at the high concentration of TPS in the culture solution (see Figure 3). Furthermore, it is likely that ROS produced by photoirradiation of TPS efficiently attack the intracellular substances in W31 cells to induce the cell death.

**Figure 5:**
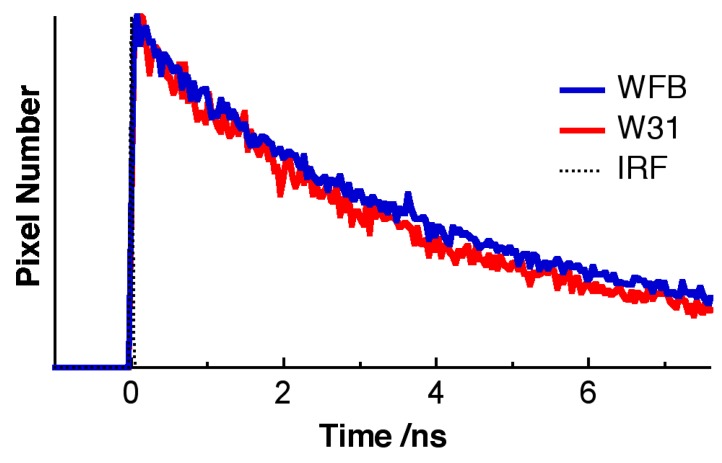
Representative fluorescence decays of TPS in WFB cells (blue) and in W31 cells (red). The scattered light is shown by a dotted line (IRF).

**Figure 6:**
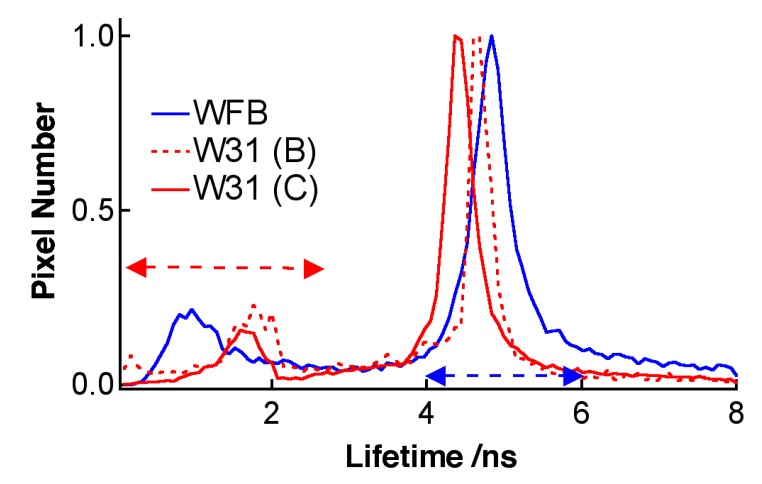
Histograms of the average fluorescence lifetime of TPS in WFB (blue) and W31 (red) cells. Red broken line and red solid line correspond to the images shown in Figure 4B and Figure 4C, respectively.

The pseudocolor of the lifetime image of TPS shown in Figure 4 is different between WFB and W31 cells, indicating that the average fluorescence lifetime of TPS in W31 cells is different from that in WFB cells. Figure 5 shows the representative fluorescence decay profiles of TPS in WFB and W31 cells, which were obtained from the FLIM measurement. These decays show that the average fluorescence lifetime of TPS is slightly shorter in W31 than that in WFB. The distribution of the average fluorescence lifetime at each pixel of the lifetime image, that is, the histogram of the fluorescence lifetime is shown in Figure 6. To obtain the lifetime image, the fluorescence decay profile at each pixel of the image was fitted by assuming a bi-exponential decay, *i.e.*, Σ*_i_A_i_*exp (–*t*/*τ_i_*), where *A_i_* and *τ_i_* are the preexponential factor and the fluorescence lifetime of the *i*th component, respectively, and the average fluorescence lifetime was given by Σ *A_i_τ_i_* (*i* = 1, 2). The peak of the distribution of the fluorescence lifetime in WFB and W31 cells are ~4.8 and 4.4 ns, respectively. These values are consistent with the fluorescence lifetime of TPS previously reported [12]. In the range of 1–2 ns, an additional peak is also observed in both cells, although the magnitude of the pixel number is small. The difference of the fluorescence lifetime reflects the difference of the intracellular environment around TPS between WFB and W31 cells, because the fluorescence lifetime is strongly affected by the environment of the fluorophore [32,33]. The change in the fluorescence lifetime of TPS with different environments results in the change in the yield of the triplet state, affecting the yield of the generation of ROS.

**Figure 7:**
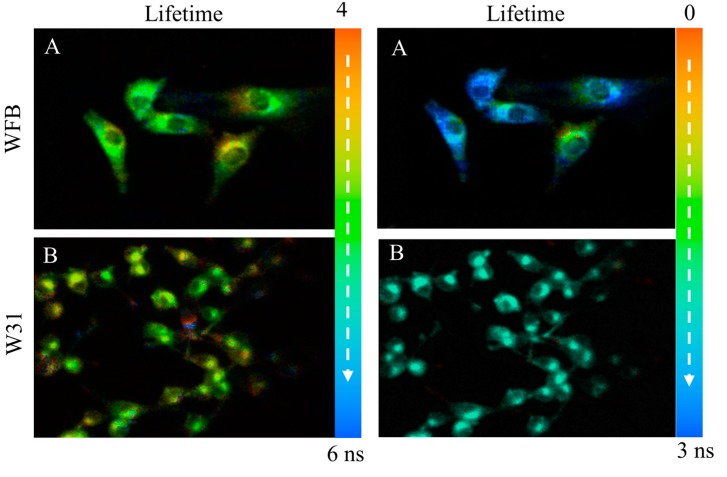
Fluorescence lifetime images of TPS in WFB cells (**upper**) and in W31 cells (**lower**) in the time range of 4–6 ns (**left**) and in the time range of 0–3 ns (**right**). These images correspond to the ones shown in Figure 4A,B, respectively. Pseudocolor is used for the lifetime images to show the value of the fluorescence lifetime.

The fluorescence lifetime of TPS was confirmed to be longer in ethanol than in PBS, as reported previously [12], suggesting that the fluorescence lifetime of TPS increases with decreasing the medium polarity. The fluorescence lifetime in both cells is shorter than that in ethanol, suggesting that TPS in cells does not significantly interact with hydrophilic substances of proteins and membranes. The distribution of the fluorescence lifetime in the image reflects the different intracellular environment around TPS within a cell and among cells [32,33]. It may be considered that the lifetime shorter than 2 ns and the lifetime longer than 4 ns, which are shown by arrows in Figure 6, correspond to the TPS whose environments are very different from each other inside cells. Note that the histogram of the lifetime images of fluorescence of TPS shows the peaks at 1.0 and 4.8 ns in WFB and the corresponding ones of W31 are 1.7 and 4.4 ns (see Figure 6). Figure 7 shows the fluorescence lifetime image of TPS in the region of the long fluorescence lifetime between 4 and 6 ns and in the region of 0–3 ns. It is clearly seen in the image of W31 cells that the long lifetime is different within a cell and among cells, which reflects the different intracellular environment from each other. The distribution of the long fluorescence lifetime is also observed in WFB cells. These results indicate that FLIM is useful to investigate intracellular environment around TPS exhibiting the photobleaching. It may be important to note that the lifetime of the long component slightly becomes shorter, as the change of the morphology is induced by photoirradiation; that is, as the cell death is induced following the ROS generation by photoirradiation. It is known that the cancer cells undergo a metabolic reprograming, resulting in altered metabolic activity. The alteration of the metabolism dysregulates intracellular pH, which may induce the difference in fluorescence lifetime of the photosensitizers between cancer cells and normal cells [34,35,36]. The presence of the local field within cells may also be one of the important factors that induce the difference in fluorescence lifetime [20,21].

**Figure 8:**
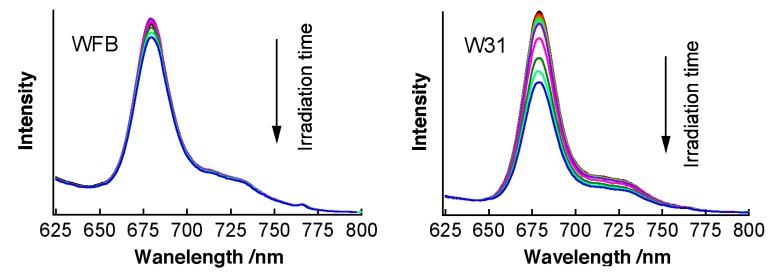
Time dependence of fluorescence spectra of Na-Ph-a in WFB (**left**) and W31 (**right**) cells with irradiation of 405-nm light in air. The irradiation times were 0, 1, 2, 3, 4, 5, 10, 20, 30 and 40 min. The concentration of Na-Ph-a in the culture solution was 10 μM.

**Figure 9:**
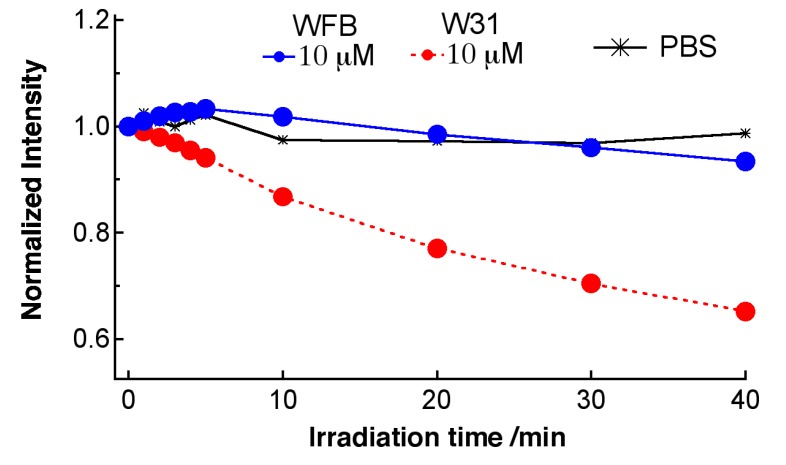
Plots of the fluorescence intensity of Na-Ph-a against the photoirradiation time in W31 cells (●) and WFB cells (●), prepared with a Na-Ph-a concentration of 10 μM. Asterisks (*) are TPS in PBS buffer. Each intensity is normalized by the intensity at 0 min.

Similar experiments have been done using Na-Ph-a as a photosentizer. Figure 8 shows time dependence of the fluorescence spectra of Na-Ph-a in WFB and W31 cells continuously irradiated at 405-nm light in air. The concentration of Na-Ph-a in the culture solution was 10 μM. The peak of the fluorescence was observed at around 670 nm and the difference in the peak wavelength between WFB and W31 cells was less than 2 nm. In both cells, the temporal decrease in the fluorescence intensity due to photobleaching was observed. The fluorescence intensity in W31 cells decreased more rapidly than that in W31 cells, as in the case of TPS. Plots of the fluorescence intensity of Na-Ph-a against the irradiation time, obtained from the spectra shown in Figure 8, are shown in Figure 9. It is clearly seen that the photobleaching of the fluorescence of Na-Ph-a efficiently occurs in W31 cells, whereas WFB cells exhibit only a slight change in the magnitude of the photobleaching. The photoinduced quenching of fluorescence of Na-Ph-a may come from the efficient generation of ROS, as mentioned above for TPS in cells. It should be mentioned that the fluorescence intensity of Na-Ph-a in PBS buffer was essentially the same during photoirradiation for 30 min.

**Figure 10:**
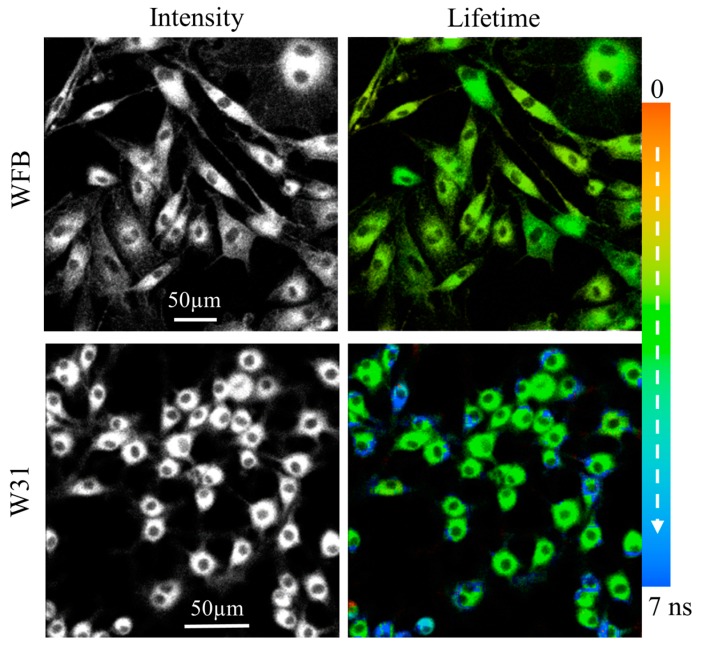
Fluorescence intensity images (**left**) and corresponding fluorescence lifetime images (**right**) of Na-Ph-a in WFB cells (**upper**) and in W31 cells (**lower**). Pseudocolor is used for the lifetime images to show the value of the fluorescence lifetime.

The fluorescence intensity and the corresponding fluorescence lifetime images of Na-Ph-a in WFB cells and in W31 cells are shown in Figure 10. The intensity reflects the concentration of Na-Ph-a at each cellular compartment. The dull round region, which is assigned to nucleus, was clearly observed in the intensity image both of W31 cells and of WFB cells, though Na-Ph-a was reported to be located in nuclei, mitochondria and lysosome of human cells [22]. In contrast with TPS-stained cells, the morphology of the Na-Ph-a stained cells is not affected by the continuous photoirradiation significantly, suggesting that not only WFB cells but also W31 cells are not so efficiently damaged by photoirradiation. ROS produced by photoirradiation of Na-Ph-a in cells may react to organic dyes efficiently, not induce the destruction of cells, resulting in the change only in the fluorescence intensity.

**Figure 11:**
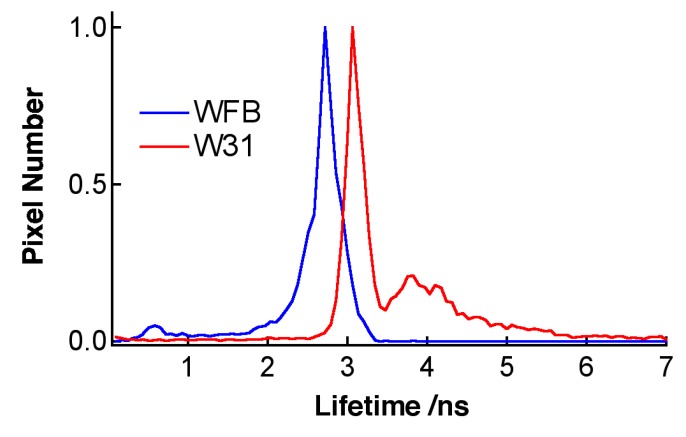
Histograms of the average fluorescence lifetime of Na-Ph-a in WFB (blue) and W31 (red) cells.

As in the case of TPS-stained cells, fluorescence lifetime of Na-Ph-a in W31 cells is significantly different from that in WFB cells. The distribution of the average fluorescence lifetime at each pixel of the lifetime image is shown in Figure 11. It is noted that the fluorescence decay profile at each pixel of the image was fitted by assuming a bi-exponential decay, to obtain the lifetime image. The peak of the distribution of the fluorescence lifetime in WFB and W31 cells is ~2.7 and 3.1 ns, respectively. The difference in the fluorescence lifetime reflects the difference in the intracellular environment of Na-Ph-a between WFB and W31 cells because the fluorescence lifetime is strongly affected by the environment of the fluorophore [32,33]. It may be important to note that the relation of the fluorescence lifetime of photosensitizer in normal and cancer cells depends on the photosensitizer; the fluorescence lifetime of Na-Ph-a is longer in cancer cells than in normal cells. The relation is the opposite in the fluorescence lifetime of TPS, regarding the long lifetime component; that is, the fluorescence lifetime of TPS is shorter in cancer cells than in normal cells. These results imply that TPS and Na-Ph-a are combined with different sites from each other in cells, though the fluorophores are porphyrin derivatives, both in TPS and Na-Ph-a.

The morphology of the Na-Ph-a stained cells is not affected by photoirradiaion significantly, which may suggest that the efficient cell death is not induced by photoirradiation in Na-Ph-a-stained cells. The cell viability with photosensitizers in normal and cancer cells depends on the concentration of the photosensitizer [37] and on the irradiation light intensity. With the present experimental system, it is not possible to estimate the exact viability values for both the photosensitizers in normal and cancer cells. From the measurements of the fluorescence intensity (see Figure 3 and Figure 9), however, it may be suggested that the viability of normal cells is much higher than that of cancer cells in both sensitizers of TPS and Na-Ph-a. It may also be true that the cell viability with Na-Ph-a is much higher in both cells than that with TPS.

## 4. Conclusions

We have measured the fluorescence spectra and the fluorescence lifetime images of TPS and Na-Ph-a in normal cells (WFB) and in cancer cells (W31). TPS-accumulated W31 cells as well as Na-Ph-a accumulated W31 cells exhibit the significant quenching of the fluorescence by photoirradiation of 405-nm light. These photoinduced phenomena can be explained in terms of the excessive generation of ROS via photoexcitation of TPS or Na-Ph-a in W31 cells. The fluorescence lifetimes, both of TPS and of Na-Ph-a, in WFB cells are different from the ones in W31 cells; the fluorescence lifetime of TPS is shorter in W31 cells than that in WFB cells, whereas the fluorescence lifetime of Na-Ph-a is longer in W31 cells than that in WFB cells, concerning the slow fluorescence component. These results show that the intracellular environments surrounding the photosensitizers in cancer cells are very different from those in normal cells, indicating that the measurement of FLIM is very useful to investigate the intracellular environment around the photosensitizers. The efficient morphological change of TPS-stained W31 cells by photoirradiation seems to imply that the photosensitizer TPS leads to more efficient death of cancer cells by photoirradiation than the other, where Na-Ph-a is used as a photosentizer, as far as W31 cells are concerned.
